# A New Global Air Quality Health Index Based on the WHO Air Quality Guideline Values With Application in Cape Town

**DOI:** 10.3389/ijph.2023.1606349

**Published:** 2023-10-23

**Authors:** Temitope Christina Adebayo-Ojo, Janine Wichmann, Oluwaseyi Olalekan Arowosegbe, Nicole Probst-Hensch, Christian Schindler, Nino Künzli

**Affiliations:** ^1^ Department of Epidemiology and Public Health, Swiss Tropical and Public Health Institute, Basel, Switzerland; ^2^ University of Basel, Basel, Switzerland; ^3^ Faculty of Health Sciences, School of Health Systems and Public Health, University of Pretoria, Pretoria, South Africa; ^4^ Department of Epidemiology and Biostatistics, School of Public Health, Imperial College London, London, United Kingdom; ^5^ MRC Centre for Environment and Health, School of Public Health, Imperial College London, London, United Kingdom; ^6^ Swiss School of Public Health (SSPH+), Zürich, Switzerland

**Keywords:** air pollution, air quality guidelines, health effects, globalized air quality health index, air quality regulations

## Abstract

**Objectives:** This study developed an Air Quality Health Index (AQHI) based on global scientific evidence and applied it to data from Cape Town, South Africa.

**Methods:** Effect estimates from two global systematic reviews and meta-analyses were used to derive the excess risk (ER) for PM_2.5_, PM_10_, NO_2_, SO_2_ and O_3_. Single pollutant AQHIs were developed and scaled using the ERs at the WHO 2021 long-term Air Quality Guideline (AQG) values to define the upper level of the “low risk” range. An overall daily AQHI was defined as weighted average of the single AQHIs.

**Results:** Between 2006 and 2015, 87% of the days posed “moderate to high risk” to Cape Town’s population, mainly due to PM_10_ and NO_2_ levels. The seasonal pattern of air quality shows “high risk” occurring mostly during the colder months of July–September.

**Conclusion:** The AQHI, with its reference to the WHO 2021 long-term AQG provides a global application and can assist countries in communicating risks in relation to their daily air quality.

## Introduction

In 2012, approximately 50 million South Africans (95%) were exposed to harmful concentrations of ambient particulate matter with aerodynamic diameter <2.5 µm (PM_2.5_) and ozone (O_3_) with measurements above the national ambient air quality standards (NAAQS) of 10 μg/m^3^ and 120 μg/m^3^, respectively [1]. In South Africa, the total burden of disease attributable to PM_2.5_ was estimated at 19,507 premature deaths, with 463,028 (95% Uncertainty interval (UI): 273,422–632,937) disability-adjusted life years (DALYs); while 1734 premature deaths due to COPD were attributed to O_3_ with 61,130 DALYs (95% UI: 25,634–84,605) [1].

The daily communication of air quality to the public has been in practice since the late nineties with the use of Air Quality Index (AQI) and lately in the early 2000s, the Air Quality Health Index (AQHI).

The AQI is conventionally developed using criteria pollutants of which the short-term average concentrations are compared to the short-term limit values set by the national ambient air quality standards (NAAQS). The pollutant with the highest value relative to its limit value determines the short-term AQI value [2]. This means the AQI is based on reporting the most offending pollutant, while ignoring the “lower levels” of the other pollutants. This is one of the core reasons why the index has received criticism. As countries adopt different NAAQS, air quality indices are not comparable across countries, which is a confusing feature of a tool adopted to communicate the risks related to daily levels of air pollution. In particular, the lowest index values are usually labeled as “green” or “healthy air”. Thus, with discrepant AQI scales, the same level of pollution may be communicated as “green” in one city or country but “hazardous” elsewhere. Other limitations of AQIs include their inability to reflect additive or combined effects of multiple pollutants, to capture effects below thresholds and that they are rarely updated when the NAAQS are reviewed or amended [3–5].

In South Africa, the NAAQS of the pollutants are less stringent than those proposed by WHO in 2005 and, thus, far less stringent than the new 2021 WHO air quality guideline (AQG) values. This has major implications on the way South Africa communicates short-term air quality to the public. South Africa’s AQI has five bands on a scale of 1–10 indicating “low,” “moderate,” “high” “very high” and “hazardous” risk levels of air quality [6]. The bands defining “good” air quality or “low” pollution are enormous, with hourly concentration of PM_2.5_, PM_10_, NO_2_, SO_2_ and O_3_ varying from 0–103 μg/m^3^, 0–190 μg/m^3^, 0–200 ppb (376 μg/m^3^), 0–350 ppb (916.7 μg/m^3^) and 0–80 ppb (157 μg/m^3^), respectively. Thus, concentrations within these ranges are declared to be “safe” or healthy although they may be far higher than the 2005 WHO Air Quality Guideline values [7]. Therefore, the misclassification of the air quality levels in this index leads to an underestimation of the true risks. In fact, only extreme episodes of unusually high levels of air pollution above NAAQS can be captured, which, in most parts of the country, are rare as seen on the South African Air Quality Information System (SAAQIS) [8].

In contrast, the health-based multipollutant indices commonly known as AQHI have the primary objective of comprehensively accounting for the short-term health effects of multiple air pollutants. The AQHI reflects the overall influence of different mixtures of air pollutants and the presence of effects at low levels of exposure, which by design is a limitation of the AQI. Cairncross et.al. constructed a health-based multipollutant index a decade before South Africa implemented the AQI. They used relative risks for daily mortality from a WHO health impact assessment conducted in Europe to illustrate the method for developing the index [3]. A well-constructed AQHI must have a few attributes as highlighted by Hewings [2]. These involve the inclusion of criteria pollutants and their synergies, expandable for other pollutants and averaging times; comparability among communities; understandability to the public; and usability as an information and alert system.

We add two other criteria that an AQI or AQHI index should fulfill. First, a health oriented index should consistently weigh the health impact of each pollutant. Second, the long-term WHO AQG values rather than the short-term values should be a point of reference to properly reflect the scientific evidence in the interpretation of short-term concentrations. WHO does not consider the short-term AQG values as a “healthy” reference but as a concentration that should not be exceeded more than three times a year. Instead, AQI ignore this statistical definition of short-term limit values but consider these concentrations as “healthy” irrespective of the number of exceedances. This results in the paradox that daily compliance with the short-term guideline values will define air quality as “healthy” although the annual mean may still be far above the long-term WHO AQG value.

In the 2021 WHO AQG update it has been emphasized, that the effect of ambient air pollution on mortality, cardiovascular and respiratory disease hospital admissions can be observed at levels lower than WHO 2005 air quality guidelines and South Africa’s NAAQS [9–15], thus, AQG values have been lowered. This calls for a revision of the AQI and we take this as an opportunity to develop a globally generalizable index that addresses the limitations and paradox of current AQI discussed above.

Therefore, this study proposes a revised methodology for the AQI to be of direct relevance for South Africa and beyond. We describe the numeric formulation of the index and its health standardized scaling, which uses the WHO 2021 long-term AQG values as point of reference to define “healthy” air quality. We also propose the translation of the scale into a traffic-color-based scheme (green-yellow–red). Finally, the constructed index is applied to daily air pollution data from Cape Town, 2006–2015.

## Methods

**FIGURE 1:**
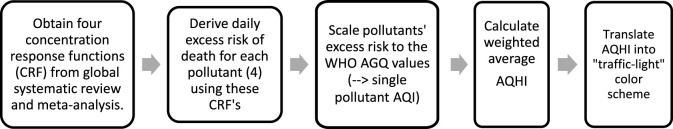
A four-step guide for constructing an Global Air Quality Health Index (AQHI) Cape Town, South Africa 2006 and 2015.

The development of our health-based multiple pollutant index which will be referred to as AQHI for simplicity requires five steps as illustrated in Figure 1. Each step is described in more detail in the method section of the Supplementary Material. In summary, the numeric formulation of the AQHI starts with using existing epidemiological concentration-response functions (CRF) for four ambient pollutants, generally a relative risk estimate (RR) per unit increase in the ambient concentrations. These RR from large reviews are used for the derivation of the new WHO AQG (2021) [16, 17]. In the second step we used these CRF’s to derive the daily excess mortality risks for each of the four pollutants. Third, we scaled the distribution of each pollutant’s excess risk (ER) to index values with linear categories from 1 to 10+ in a way that the index value of 3 corresponds to the ER derived for the concentrations where the WHO long-term AQG values are met. Fourth, the overall AQHI is calculated by taking the weighted average of the four index values. In the last step, we categorize the 10 index units into the color scheme of traffic lights where “green” will be up to level 3 of the scale, thus in compliance with the excess risk occurring at concentrations up to the long-term WHO AQG values of each pollutant. Therefore, if concentrations of all pollutants remain on all days within the “green” levels, air quality will also be compliant with the long-term AQG values. Concentrations above the index value of 10 all fall into the unbounded upper category of “10+”.

In the last section, we will apply the new AQHI to the time series of Cape Town used in the first step to demonstrate the features of the AQHI and the level of compliance of the past air quality in Cape Town with the proposed index.

Due to the high correlation between PM_10_ and PM_2.5_, and given that some authorities restrict the monitoring of PM to only one fraction, we propose to derive the AQHI with either one of the two size fractions of PM. Thus, each of the two AQHI will include four pollutants, namely the three gaseous pollutants but only one of the two particulate mass fractions. In our case study, we will apply the PM_10_ based AQHI to our 2006–2015 Cape Town data.

The daily ERs were calculated using Eq. 1, therefore, the excess risk associated with the long-term WHO AQG-value c_i_ of pollutant i becomes $100\left(e^{\beta_{i}c_{i}}-1\right)$ .

$$pollutant\;i\;excess\;risk\;on\;day\;t=100\left(e^{\beta_{i}x_{i}\left(t\right)}-1\right)$$ (1)

$$\beta_{i}=coeffcient\;per\;1\frac{ug}{m3}increase\;of\;pollutant\;i,x_{i}\left(t\right)=concentration\;of\;pollutant\;i\;on\;day\;t)$$

We used the ERs associated with an index of 1 (Table 1) to define the weights of the pollutant-specific AQHIs in the overall AQHI. For each pollutant i, the weight W_i_ is defined as the ratio between the ER of PM_10_ (or PM_2.5_) and the ER of the pollutant i. Thus, the weight of PM_10_ (or PM_2.5_) is defined to be 1. The daily average AQHI value is the weighted mean of the index values of the different pollutants using Eq. 2, rounded to the nearest integer.

$$Weighted\;Average\;AQHI\left(t\right)=\frac{1}{\sum W_{i}}\sum_{i=1\ldots n}W_{i}{*AQHI}_{i}\left(t\right)$$ (2)

$$where\;n=number\;of\;pollutants\;used\;in\;AQHI,i=pollutant,{AQHI}_{i}\left(t\right)=derived\;index\;value\;for\;pollutant\;i\;on\;day\;t\;and\;W_{i}=weight\;of\;{AQHI}_{i}\left(t\right)$$

**TABLE 1:** Derivation of the weighted average AQHI indices: the single pollutant concentration-response functions (CRF), the related beta coefficient, the chosen WHO AQG reference value, [16, 17] the related daily excess risk (ER) (Eq. 1). In addition, the daily ER%s of the pollutants ER% per index unit are shown. Thus, by design, the single pollutant index value of 3 corresponds to PM_10_, NO_2_, SO_2_ and O_3_ concentrations of 15 μg/m^3^, 10 μg/m^3^, 20 μg/m^3^, and 60 μg/m^3^, respectively. The weights for the average index value are shown for both, the PM_2.5_ and the PM_10_ based AQHI. Cape Town, South Africa 2006 and 2015.

Pollutant p	CRF published in WHO AQG (per 10 μg/m^3^)	Beta coefficient per 1 μg/m^3^	WHO AQG reference value [1] in µg/m^3^ for index value = 3	ER (%) at index value = 3	Average ER (%) per index unit	Inverse weight for PM_2.5_ based AQHI	Inverse weight for PM_10_ based AQHI
PM_2.5_	1.0065	0.00065	5	0.326	0.109	1	—
PM_10_	1.0041	0.00041	15	0.617	0.206	—	1
NO_2_	1.0072	0.00072	10	0.723	0.241	0.451	0.853
SO_2_	1.0059	0.00059	20^2^	1.187	0.396	0.275	0.519
O_3_	1.0043	0.00043	60	2.614	0.871	0.125	0.236

Given that monitoring stations may occasionally not be functional, authorities will face the challenge of missing data. We propose a simple imputation in the Supplementary Material. Otherwise, the weighted average AQHI may be based on less than four index values.

Using the result from Table 1 we present the final AQHI in Table 2 below:

**TABLE 2:** The constructed AQHI showing the range of excess mortality risk per pollutant, levels of risk and the corresponding health messages. Cape Town, South Africa 2006 and 2015.

	Single pollutant ER% range					Health messages	
AQHI	PM_10_	NO_2_	SO_2_	O_3_	Risk levels	General population	Susceptible population
1	<0.21	<0.24	<0.4	<0.87	Low risk (AQHI 1–3)	Ideal conditions for regular outdoor activities	Enjoy your usual outdoor activities
2	>0.21–0.42	>0.24–0.48	>0.4–0.8	>0.89–1.74			
3	>0.42–0.63	>0.48–0.72	>0.8–1.2	>1.74–2.61			Follow your doctor’s advice for exercise
4	>0.63–0.84	>0.72–0.96	>1.2–1.6	>2.61–3.48	Moderate risk (AQHI 4–6)	No need to modify your usual outdoor activities	If you have heart or breathing problems, and experience symptoms, consider reducing physical exertion outdoors or rescheduling activities to times when the index is lower
5	>0.84–1.05	>0.96–1.20	>1.6–2	>3.48–4.35			Contact your doctor and follow their advice
6	>1.05–1.26	>1.20–1.44	>2–2.4	>4.35–5.22			
7	>1.26–1.47	>1.44–1.68	>2.4–2.8	>5.22–6.09	High risk (AQHI 7–10+)	Consider reducing or rescheduling strenuous outdoor activities to periods when the index is lower, especially if you experience symptoms	Children, the elderly and people with breathing or heart problems should avoid physical exertion outdoors
8	>1.47–1.68	>1.68–1.92	>2.8–3.2	>6.09–6.96			
9	>1.68–1.89	>1.92–2.16	>3.2–3.6	>6.96–7.83			If you have heart or breathing problems, follow your doctor’s advice about managing your condition
10+	>1.89–2.10+	>2.16–2.40+	>3.6–4.0+	>7.83–8.70+			

### Application of the Proposed Method to Cape Town

In this section we used the daily air pollution monitoring data from Cape Town from 2006–2015 which was aggregated to city level from all available stations and analyzed for previous publications [10, 11]. We described the distribution of daily concentrations of each pollutant and of the daily ER% in this long-term time-series. In addition, the total daily ER% was translated into the pollutant-specific daily index values. In the last step, we derived the daily weighted average AQHI, based on PM_10_ and the three gaseous pollutants, as described in the Methods.

## Results

The daily averages (standard deviation) of PM_10_, NO_2_, SO_2_ and O_3_ were 30.4 μg/m^3^ (13.6 μg/m^3^), 17 μg/m^3^ (8.8 μg/m^3^), 11 μg/m^3^ (5.5 μg/m^3^) and 33 μg/m^3^ (12.3 μg/m^3^), respectively. These data have been previously described in detail [10]. The 2021 WHO short-term air quality guideline values were exceeded on 497 (13.6%) days of the 3,652 day study period for PM_10_ (>45 μg/m^3^), 501 (13.7%) days for NO_2_ (>25 μg/m^3^), and 196 (5.4%) days for SO_2_ (>40 μg/m^3^); however we did not observe any exceedance for Ozone (>100 μg/m^3^). The daily concentrations of PM_10_ and NO_2_ exceeded the WHO AQG 2021 long-term values on 93% (*n* = 3,399) and 70% (n = 2,533) of the days of the study period. The daily means of each pollutant during the study period of 2006–2015 are shown in (Supplementary Figure S2). Ozone levels after 2010 were below the WHO AQG long-term value while PM_10_ shows a decreasing trend. NO_2_ and SO_2_ do not show a discernible trend.

The highest average daily excess risk (ER%) was observed for PM_10_ with an ER% of 1.25%, while SO_2_ had the lowest ER% with a daily average of 0.6%; NO_2_ and O_3_ averaged 1.08% and 1.05% respectively. The number of days on which the individual AQHIs were in agreement with the long-term values of the WHO 2021 AQG, i.e. with an AQHI of 1, 2 or 3 and a “green” color code, was 277 (7.58%), 741 (20%), 3,366 (92.17%) and 2,613 (71.55%) for PM_10_, NO_2_, SO_2_ and O_3_, respectively. The distribution of the individual pollutants and their AQHIs is shown in Table 3.

**TABLE 3:** Distribution of daily mean (standard deviation) concentration of pollutants and number of days per weighted average-AQHI value in Cape Town for the period from 2006 to 2015 (in total, 3,652 days) Cape Town, South Africa 2006 and 2015.

Single -AQHI	PM_10_		NO_2_		SO_2_		O_3_	
	µg/m^3^	Days	µg/m^3^	Days	µg/m^3^	Days	µg/m^3^	Days
1^3^	—	—	—	—	8.3 (4.7)	3 (0.1%)	—	—
2	15.1 (3.2)	33 (0.9%)	3.9 (1.4)	4 (0.1%)	7.2 (2.9)	30 (0.8%)	30.8 (10.3)	24 (0.7%)
3	19.3 (5.6)	419 (11.4%)	7.2 (2.8)	265 (7.3%)	8.0 (4.1)	398 (10.9%)	32.4 (10.9)	308 (8.4%)
4	22.5 (7.8)	1,015 (27.8%)	11.0 (3.8)	916 (25.1%)	8.9 (4.3)	984 (26.9%)	30.9 (12.6)	715 (19.6%)
5	29.1 (10.2)	954 (26.1%)	15.3 (5.1)	875 (24.0%)	10.0 (4.6)	945 (25.9%)	33.9 (12.5)	720 (19.7%)
6	36.2 (10.9)	602 (16.5%)	18.7 (6.1)	601 (16.5%)	11.5 (5.3)	598 (16.4%)	35.2 (12.3)	440 (12.0%)
7	42.8 (11.3)	345 (9.4%)	24.6 (6.6)	345 (9.4%)	12.6 (4.7)	337 (9.2%)	34.7 (12.4)	249 (6.8%)
8	51.6 (11.0)	241 (6.6%)	32.8 (7.1)	214 (6.6%)	16.6 (6.3)	241 (6.6%)	32.5 (11.4)	181 (5.0%)
9	65.1 (13.2)	37 (1.0%)	41.8 (8.3)	37 (1.0%)	26.8 (5.3)	37 (1.0%)	30.7 (11.3)	35 (1.0%)
Missing	—	9(0.3%)	—	368 (10.1%)	—	79(2.2%)	—	980 (26.8%)

AQHI level 3 indicates PM_10_ exceeds on average the WHO long-term value (15 μg/m^3^ vs. 19 μg/m^3^) while the means of the other pollutants are below their long-term WHO AQG values. PM10, with the lowest number of missing days (0.2%) and contributing more weight to the combined index, likely compensated for missing measurements of other pollutants.

**FIGURE 2:**
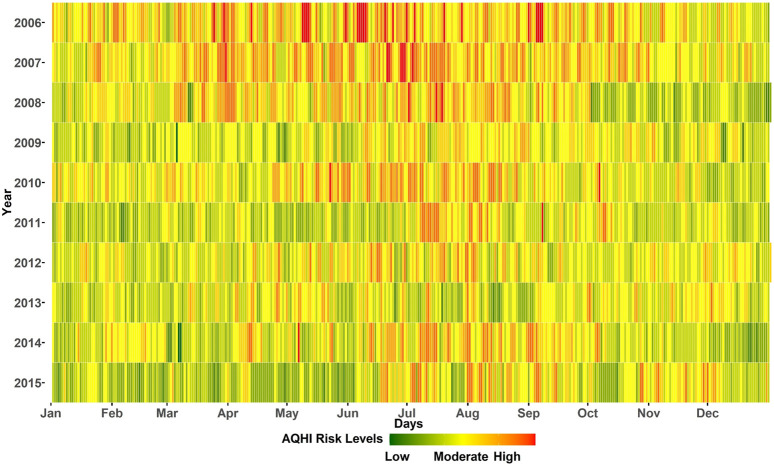
Daily global air quality health index. Colors correspond to the proposed “traffic light categories” of the AQHI. Cape Town, South Africa 2006 and 2015.

Figure 2 shows the air quality in Cape Town. The weighted average AQHI for the combination of all four pollutants during the study period of 3,652 days was “low risk” on 482 days (13%), “moderate risk” on 2,565 days (70%) and“high risk” on 605 days (17%). In the first 2 years, there were 6 “low risk” days each and the last year (2015) had the highest number of “low risk” days (123 days, i.e.33%). There appears to be an improvement in air quality when comparing the beginning and the end of the study period, but there was no clear trend, as the number of “low risk” days varied in the intervening years. In addition, the first 3 years had more “moderate-high risk” days between April and September. After 2009, however, the seasonal pattern became more pronounced with “high risk” days occuring mostly in the colder months of June–September. We provide an interactive plot showing the single pollutant AQHIs and the weighted average AQHI for the study period in Cape Town, South Africa 2006 and 2015.

## Discussion

This study constructed a globally applicable Air Quality Health index using concentration-response functions (CRF) obtained from recent global systematic reviews on the short-term effects of air pollutants on daily mortality [16, 17]. It is the first index to incorporate the newly published long-term WHO Air Quality Guideline values as a reference point to define “healthy” or “low risk” days. Thus, judgments about daily air quality will not contradict current evidence of health effects occurring at concentrations exceeding the long-term AQG values. Indeed, all AQI currently in use can lead to the paradox that all daily means may be labeled “green” or healthy although the annual mean may substantially exceed the WHO reference values.

Our novel index keeps a methodological similarity with the Canadian AQHI. The latter index was constructed with an assumption of linear, no-threshold associations between the exposure to air pollutants and daily excess mortality. The appropriateness of this approach was also demonstrated in recent systematic reviews, including a particularly large multicity study on particulate matter and daily mortality also used in the derivation of the new WHO AQG values [18].

The application of our index to data from Cape Town showed that the proposed AQHI would qualify 87% of the days in our study period as “moderate” or “high risk”. This strongly contradicts the risk levels communicated via the current South African AQI where the past years would mostly be labeled as “good”. A large body of literature endorses the revised qualification of Cape Town’s air quality. Previous studies of short-term effects of air pollution on cardiorespiratory health in the study area reported that PM_10_ and NO_2_ were positively associated with hospital admissions and at levels far below the average daily concentrations observed in Cape Town during the study period. An interquartile range (IQR) increase of 12 μg/m^3^ for PM_10_ and 7.3 μg/m^3^ for NO_2_ were associated with a 2% (95% confidence interval (CI): 0.5%–3.2%) and 2.3% (95% CI: 0.6%–4%) increased risk of respiratory disease hospitalizations, respectively [11]. In addition, the same increment in PM_10_ was associated with a 2.1% increased risk in cardiovascular hospitalization [11]. Another study on CVD and RD mortality showed a 4.5% increased risk of CVD mortality (95% CI: 1.4%–7.6%) for an IQR change of 10.7 μg/m^3^ in NO_2_. In addition, an IQR change of 16 μg/m^3^, 11 μg/m^3^, and 16 μg/m^3^ in PM_10_, NO_2_ and O_3_ was associated with an increased risk of 2.4% (95% CI: 0.9%–2.2%), 2.2% (95% CI: 0.4%–4.1%) and 2.5% (95% CI: 0.2%–4.8%) in RD mortality, respectively [10]. During our study period, the ER% for the average PM_10_ (30.3 μg/m^3^) and NO_2_ (16.6 μg/m^3^) levels would correspond to 1.25 ER% (AQHI 10) and 1.2ER% (AQHI 6) of death, respectively. Thus, it is appropriate to label the air quality to which the population of Cape Town was exposed to as poor rather than as “low risk”.

There is no universal method for constructing an AQHI; most authors have developed their index using the methods of Cairncross and Stieb, but the indices differ in the number of pollutants, averaging times and breakpoints for risk classification [3, 5]. Our use of established effect estimates is similar to Cairncross’ air pollution index, but these authors used estimates from a European study whereas ours are from a global systematic review. Ideally, an AQHI would communicate the combined effects of the pollution mixture. The approach of Stieb et.al. [5] to develop an AQHI based on multi-pollutant time-series analyses, was indeed an intriguing proposal along these lines. However, the number of multi-pollutant studies is very limited, thus, the derivation of mutually adjusted effect estimates would rely on thin data, usually from high income countries. Moreover, most multipollutant studies evaluated only two-pollutant models whereas mutually adjusted models with three or even all four pollutants used in our AQHI are not available [16]. Thus, we consider our approach based on single-pollutant CRFs as adequate.

Our approach challenges though the derivation of a combined AQHI summary measure. If the four AQHI were based on mutually adjusted CRF’s, the sum of the four estimates would be an adequate measure of the overall AQHI. However, the sum of single-pollutant ERs would clearly overestimate the true total ER given the substantial correlation between single pollutants such as PM and NO_2_ or SO_2_. Without proper knowledge of the degree of overlap it is impossible to properly adjust the sum of single-pollutant based ERs. Thus, to nevertheless integrate information of four pollutants into one single AQHI, we derived a weighted average AQHI. Inevitably, this will underestimate the total risk to the extent that at least part of the effects of single pollutants are additive, i.e. independent of those estimated for the other pollutants. Indeed, for PM and ozone, risk assessors agreed to treat those as independent effects, thus, the Global Burden of Disease integrates the sum of both into the assessment of the total air pollution related burden [19]. Instead for the other three pollutants, combined models are not yet available. In fact, a recent study made valuable first efforts to integrate mutually adjusted risk estimates for two pollutants, namely, PM_2.5_ and NO_2_ [20].

As mentioned, a novelty of our AQHI is the full alignment with the WHO AQG values. AQHI values 1 to 3 (green) all comply with daily concentrations up to the long-term mean guideline values. Our method reveals an interesting feature of the AQG values, which plays a key role in the derivation of the overall average index value. As emphasized in the WHO AQG (2021) [9], the Guideline Development Group did not define any “acceptable” health burden to derive the guideline values. Instead the lowest concentration for which effects could be observed with sufficient confidence were taken to define the long-term AQG values. This contrasts with the prevailing risk management concept for carcinogens where “acceptable risks”—e.g., 1 case per 1 Million lives—are defined as “acceptable” policy target [21]. The WHO AQG emphasize also the lack of evidence for any “thresholds of no effect” for the pollutants used in the AQHI, thus, concentration below the guideline values are not considered “healthy” but the shape of the CRF is not yet defined below those levels. If one estimates the excess risk for the concentrations proposed by WHO as the guideline values as compared to zero pollution, one obtains in essence the implicitly defined “acceptable risks” as shown in Table 1. Those ER vary substantially across the four pollutants. E.g. the ER% at the limit value of ozone is 4.23 times higher than the ER% at the new guideline value of PM_10_. In other words, the WHO AQG has the inherent inconsistency of tolerating a much higher health burden due to ozone than due to PM_10_. Thus, taken at the same index level (e.g. 3), the arithmetic mean of four ER% would be dominated by the burden due to ozone.

As a consequence of the dominance of the ER% scaling of ozone and of the much more likely compliance of ozone with the AQG values the arithmetic mean of the four index values would often mask “high risk” days of PM_10_ (and NO_2_) as “low risk” days. Such bias jeopardizes the intention of the AQHI, namely to coherently communicate the daily health risks due to air pollution. Thus, instead of using the arithmetic mean we derive the weighted mean AQHI using the inverses of the ER% at the WHO AQG reference values as the weights. As shown in Table 3, as a consequence of this weighting, the measured concentrations of the four pollutants are mostly below the long-term AQG values on days when the derived overall AQHI results at level 1, 2 or 3. However, in case of PM10 the long-term AQG value is exceeded on 296 days (8%) of the study period partly due to its weight.

Our proposal for a globally adopted AQHI is an innovative approach as it offers a fresh perspective on the long-standing issues of AQIs. It fully standardizes the science based communication of risk levels irrespective of the local policies and pollution. It endorses the “right to know” on a global scale, in an equitable manner. On the other side, it forces authorities in regions with very high levels of air pollution to label air quality on most if not all days as “red” or “high risk.” Globally harmonized AQHI facilitate the comparison of air quality across geographical locations (within or between countries). A standardized index could provide additional value in tracking air quality trends over time, which can help authorities to evaluate their efforts and policies to achieve clean air.

For the reporting of the AQHI, authorities may adopt various approaches. The index could be reported for each monitoring station or for the mean values of each pollutant across all stations of a geographical location. Such regional mean AQHI could also help to reduce exposure misclassification as people are exposed at different levels of air pollution as they move within the region (e.g. for work). Authorities may also opt for the reporting of all four single-pollutant AQHI and the related weighted average. This would transparently disclose problematic pollutants. However, for the users of the AQHI, it may be confusing to deal with five different values. Thus, the reporting of the weighted average AQHI might be the preferable choice. In addition, sensitivity analysis of PM_10_-and PM_2.5_-based indices and simple imputation for missing pollutants are discussed in the Supplementary Material.

We propose to replace currently used AQI with our new scheme. The communication messages of current AQI are, however, still adequate (see Table 2). As shown in the literature [5], the communication of AQI values and related health information assists the general population to keep track of the air quality and possibly subscribe to receiving notifications for when the risk level exceeds a certain threshold, e.g., when it goes beyond green for people at risk. A study in Canada showed that air quality alert programs led to a 25% (95%CI 1%–47%) reduction in asthma-related emergency department visits [22]. Another study in Chile, reported a reduction in deaths among the elderly (age >64 years) following the announcement of above-average pollution episodes; the Chilean authorities accompanied their announcements with mandatory measures such as driving restrictions to reduce car emissions, shutting down of certain large stationary emitters, and other protocols, which resulted in a further 20% reduction in air pollution compared to days without alerts [23]. This shows that mandatory measures, such as those implemented in Chile, could be more effective in reducing pollution and protecting human health if accompanied by air quality alert at a certain threshold—for example, when the AQHI risk level approaches “high risk.” We recognize though, that making people aware of their air quality and the associated risks may not be sufficient to change their behaviour. At the very least, it could help susceptible people to self-calibrate if they understand the levels of the index at which they experience symptoms and discomfort.

### Conclusion

This study has constructed a global air quality health index as an effective tool for communicating air quality to the public on a daily basis. The alignment of our index scale with the science based excess risks attributable to the daily concentrations of the four pollutants used in our index guarantees global comparability of local air quality levels and fosters a coherent understanding of the related health effects. This, in turn, may foster public support for the adoption of stringent clean air policies.
