# Supplementary material for: A New Global Air Quality Health Index Based on the WHO Air Quality Guideline Values With Application in Cape Town
Source: Int J Public Health. 2023 Oct 23;68:1606349. doi: 10.3389/ijph.2023.1606349 (PMC10625908; doi:10.3389/ijph.2023.1606349)
Supplement: Supplementary file 1 [file DataSheet1.docx]

# A new global air quality health index based on the WHO Air Quality Guideline Values with application in Cape Town.

Temitope Christina Adebayo-Ojo^1,2^, Janine Wichmann^3^, Oluwaseyi Olalekan Arowosegbe^4,5^, Nicole Probst-Hensch^1,2^, Christian Schindler^1,2^, Nino Künzli^1,2,6^

^1^ Department of Epidemiology and Public Health, Swiss Tropical and Public Health Institute, Basel, Kreuzstrasse 2, 4123 Allschwil, Switzerland;

^2^ University of Basel, Basel, 4056, Switzerland

^3^ Faculty of Health Sciences, School of Health Systems and Public Health, University of Pretoria, Pretoria, 0002, South Africa

^4^ Department of Epidemiology and Biostatistics, School of Public Health, Imperial College London, London, UK;

^5^ MRC Centre for Environment and Health, School of Public Health, Imperial College London, London, UK

^6^ Swiss School of Public Health (SSPH+), 8001 Zürich, Switzerland

temitope.adebayo@swisstph.ch; janine.wichmann@up.ac.za; o.arowosegbe@imperial.ac.uk (O.O.A.); nicole.probst@swisstph.ch (N.p.-H.); christian.schindler@swisstph.ch (C.S.); nino.kuenzli@swisstph.ch (N.K.)

*Corresponding: temitope.adebayo@swisstph.ch

Conflict of interest: The authors declare they have nothing to disclose.

This document outlines the methods used for constructing the air quality health index (AQHI) in detail and presents additional results.

Online supplementary material

Method

The construction of the AQHI follows five steps as shown in Figure S 1. The detail of each step is outlined below.


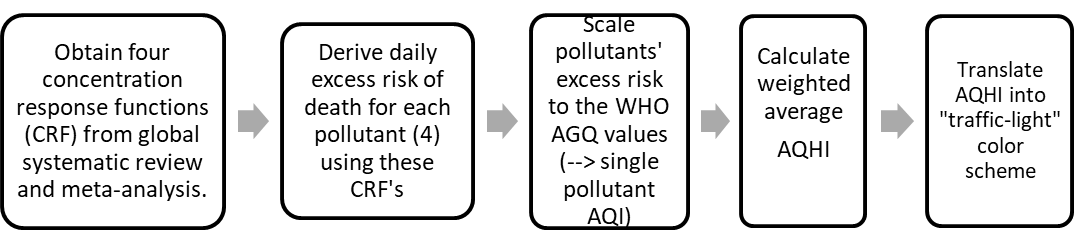


Figure S 1: A four-step guide for constructing an Air Quality Health Index (AQHI)

**Obtain concentration response functions (CRF)**

Many CRF for the association between daily mean concentrations and mortality are available. We opted for the CRFs from the global systematic review of 263 studies for PM_2.5_, PM_10_, NO_2_, SO_2_, and O_3_ and all-cause mortality that guided the development of the 2021 WHO AQG values.^1,2^ These reviews published pooled effect estimates from single-pollutant analyses. Two of the studies in the review were large multi-city studies of 652 and 406 cities, six of which were South African, conducted by the Multi-country-multi-city (MCC) collaborative research network.^3,4^ These estimates are considered to be more stable than those reported from single city study analyses and are thus suitable for constructing an index for global use. The CRFs from the review are shown in Table 1 of the main manuscript, repeated here also in Table S 1.

**Derivation of daily excess risk for each pollutant**

Next, we used these all-cause mortality CRF coefficients shown in Table S 1 to derive the excess risk (ER) for each pollutant in our study. As first point of reference for this ER we used the concentrations of each pollutant that corresponds to the WHO AQG long-term values published in 2021.^5^ Given the WHO AQG 2021 methodology, air quality is expected to be complying with those long-term values if the short-term values defined by WHO AQG are not exceeded more than three times a year.

In case of SO_2_, 2021 WHO AQG did not propose a long-term value but only the daily mean of 40µg/m^3^ not to be exceeded more than three times a year. Thus, WHO AQG have not derived the statistical relationship between the number of exceedances and the annual mean for SO_2_ as in case of the other pollutants. However, in the second edition of the WHO AQG (2000), an annual mean value of 20 µg/m^3^ was proposed to protect natural ecosystems.^6^ The value has been scientifically derived as critical load to protect the vegetation in the long run.^7^ Many legislators require environmental conditions that protect human health, vegetation, crops and animals alike. Thus, we consider this a suitable point of reference for the derivation of the ER and assume that compliance with this annual mean to correspond to only a few daily means above the WHO AQG 2021 value.

We calculated the excess risk associated with each pollutant i’s coefficient $\beta_{i}$ where $c_{i}$ is the long-term concentration defined in the WHO AQG as shown in Equation S 1.

|  | $pollutant i excess risk on day t=100\left( e^{\beta_{i}c_{i}}-1 \right)$ | Equation S 1 |
| --- | --- | --- |

$$\beta_{i}=coeffcient per 1\frac{ug}{m3}increase of pollutant i, c_{i}\left( t \right)= long-term WHO AQG concentration of pollutant i )$$

PM_2.5_ standards were implemented in 2012 and PM_2.5_ measurements were introduced only in 2018 according to SAAQIS report with more stations added in 2019 and 2020.^8^ Thus, the AQHI in this study uses PM_10_ data from 2006 – 2015. However, the derivation of a PM_2.5_ based AQHI is identical.

**Scaling the pollutant excess risk in context of the WHO AQG values**

In line with concepts of previous AQI or AQHI, our linear scale ranges from 1 to 10+. For each pollutant, the percent increase per unit of the index was chosen in such a way that the index value of 3 corresponds to the ER at the WHO long-term reference values as derived above. The ER per 1 unit index as shown in Table 1 were rounded to two decimal places and used to produce categories for 10 index values. The daily ER% of each index value, thus, corresponds for each pollutant to the ranges as presented in Table 2. For example, a PM_10_ index value of 1 corresponds to up to 0.21% excess mortality risk whereas larger ER% up to 0.42% are contained in level 2 and so on.

**Calculate overall AQHI**

The previous step derived a daily index value for each pollutant. For communication purposes, it would be informative to provide one single index value that summarizes the impact across pollutants. The sum of the four estimates is not a valid summary measure of the impact as it would assume fully independent excess risks attributable to each pollutant. Although this may be defendable for O_3_ and PM, there is agreement that single-pollutant CRFs capture partly correlated, thus, overlapping effects of e.g. PM and NO_2_ or SO_2_.^9^ However, at this stage this overlap is not well defined given the paucity of estimates of multipollutant CRFs.

Instead we propose the derivation of an “average index value” to reflect the expected daily mean health impact of the pollutants used in the index. However, the arithmetic mean of the single values has problematic features. As shown in Table 1, although the WHO AQG values are derived to protect health, the mortality ER% at the long-term AQG value grossly varies across the pollutants. Moreover, as shown in the case study later on, the ability to comply with the AQG values differs substantially across the pollutants used in the index. E.g., the new PM_2.5_ AQG are extremely ambitious for most regions in the world, compliance with the SO_2_ AQG may already be achieved in many places. Thus, the arithmetic mean of the four AQI values and the average excess risk – would be unequally influenced by this inherent discrepancy. Therefore, we propose a weighted average index value to harmonize the discrepant ER% at the WHO AQG levels. Methodological details are shown in the main text.

**Translate AQHI into "traffic-light" colour scheme**

In the last step, the AQHI is constructed by using the scaled index and translating it into “traffic-light” colour scheme. The levels of risk in the index ranges from 1 to 10+. We define “low risk” or “green” as 1 – 3, “moderate risk” or “yellow” as 4 – 6, and “high risk” or “red” as 7 – 10+. Authorities might communicate the colours for each single pollutant AQHI or the derived weighted average or both. This is a simple communication tool to enable the public understand the continuum between healthy and unhealthy air quality. The AQHI is also accompanied with health messages for the general population and population at risk, which is presented in Table 2. By design, if all four pollutants complied with the WHO AQG reference value shown in Table 1, the overall AQHI for such day will be “green”.

The health messages corresponding to the risk levels were adapted from the Canadian AQHI^10^*;* the Canadian authors were methodical in the process of developing the communication material which involved multiple stakeholders and audiences. The materials were tested and evaluated through qualitative interviews to assess knowledge, attitudes and behaviors related to air pollution and particularly to the air quality index. Therefore, we consider these messages to be well suited for our constructed AQHI. However, we did not consider the index value 10+ as separate category and therefore did not distinguish between “very high” and “high” risk.

**Sensitivity analysis**

As mentioned, we propose to build the AQHI either with PM_10_ or PM_2.5_. The question arises whether these two options would lead to the same result. To assess the sensitivity of the index for choosing between the two options, we used 2019 data from Tableview monitoring station as we had both PM_10_ and PM_2.5_ data for this year. In total, 83.5%, 90%, 97% and 97% of daily values of SO_2_, NO_2_, PM_10_ and PM_2.5_, respectively, were available for 2019. In addition, we used the O_3_ data from Atlantis where 71.5% of the data were available. This is an urban background station and thus provides an adequate estimation for the general O_3_ conditions. For the days with missing concentrations for these pollutants, their values were also missing at other stations, thus we could not perform the simple imputation as proposed in this supplementary material. In total, data for at least two gases and both PM_10_ and PM_2.5_ were available for 330 days in 2019. The total ER% was calculated for those days, separately for PM_10_ and PM_2.5_. First, we assigned the index values to each day of these two time series and the gases based on the daily ER%. Thereafter, the weighted average AQHI was derived for the PM_10_ and the PM_2.5_ based AQHI as shown in Table S1. The linear association between the PM_10_- and PM_2.5_ based weighted average AQHI was assessed in addition with the Spearman correlation coefficient.

Results

Table S 1: Derivation of the weighted average AQHI indices: the single pollutant concentration-response functions (CRF), the related beta coefficient, the chosen WHO AQG reference value, the related daily excess risk (ER) (Equation S 1). In addition, the daily ER%s of the pollutants ER% per index unit are shown. Thus, by design, the single pollutant index value of 3 corresponds to PM_10_, NO_2_, SO_2_ and O_3_ concentrations of 15 µg/m^3^, 10 µg/m^3^, 20 µg/m^3^, and 60 µg/m^3^, respectively. The weights for the average index value are shown for both, the PM_2.5_ and the PM_10_ based AQHI.

| Pollutant p | CRF published in WHO AQG (per 10 µg/m^3^) | Beta coefficient per 1 µg/m^3^ | WHO AQG reference value ^[[1]](#footnote-1)^ in µg/m^3^ for index value = 3 | ER (%) at index value = 3 | Average ER (%) per index unit | Inverse weight for PM_2.5_ Based AQHI | Inverse weight for PM_10_ Based AQHI |
| --- | --- | --- | --- | --- | --- | --- | --- |
| PM_2.5_ | 1.0065 | 0.00065 | 5 | 0.326 | 0.109 | 1 | - |
| PM_10_ | 1.0041 | 0.00041 | 15 | 0.617 | 0.206 | - | 1 |
| NO_2_ | 1.0072 | 0.00072 | 10 | 0.723 | 0.241 | 0.451 | 0.853 |
| SO_2_ | 1.0059 | 0.00059 | 20^[[2]](#footnote-2)^ | 1.187 | 0.396 | 0.275 | 0.519 |
| O_3_ | 1.0043 | 0.00043 | 60 | 2.614 | 0.871 | 0.125 | 0.236 |

The CRFs published in the reviews commissioned by WHO^1,2^ estimated risks using the 2-day mean of the pollutant measurements. We found a strong positive correlation (r > 0.87) between the daily measurements and the 2-day means of each pollutant. Thus, we propose to derive the AQHI based on daily mean data. This is easier to implement and less affected by missing data than the reliance on measurement series of 2-day means.

Table S 2: The constructed AQHI for PM2.5 showing the range of excess mortality risk and risk levels.

| AQHI | 1 | 2 | 3 | 4 | 5 | 6 | 7 | 8 | 9 | 10 |
| --- | --- | --- | --- | --- | --- | --- | --- | --- | --- | --- |
| ER% range | <0.11 | >0.11- | >0.22- | >0.33- | >0.44- | >0.55- | >0.66 | >0.77- | >0.88- | >0.99- |
|  |  | 0.22 | 0.33 | 0.44 | 0.55 | 0.66 | -0.77 | 0.88 | 0.99 | 1.10+ |
| Risk level | Low risk  AQHI 1 – 3 | | | Moderate risk  AQHI 4 – 6 | | | High risk  AQHI 7 – 10+ | | | |


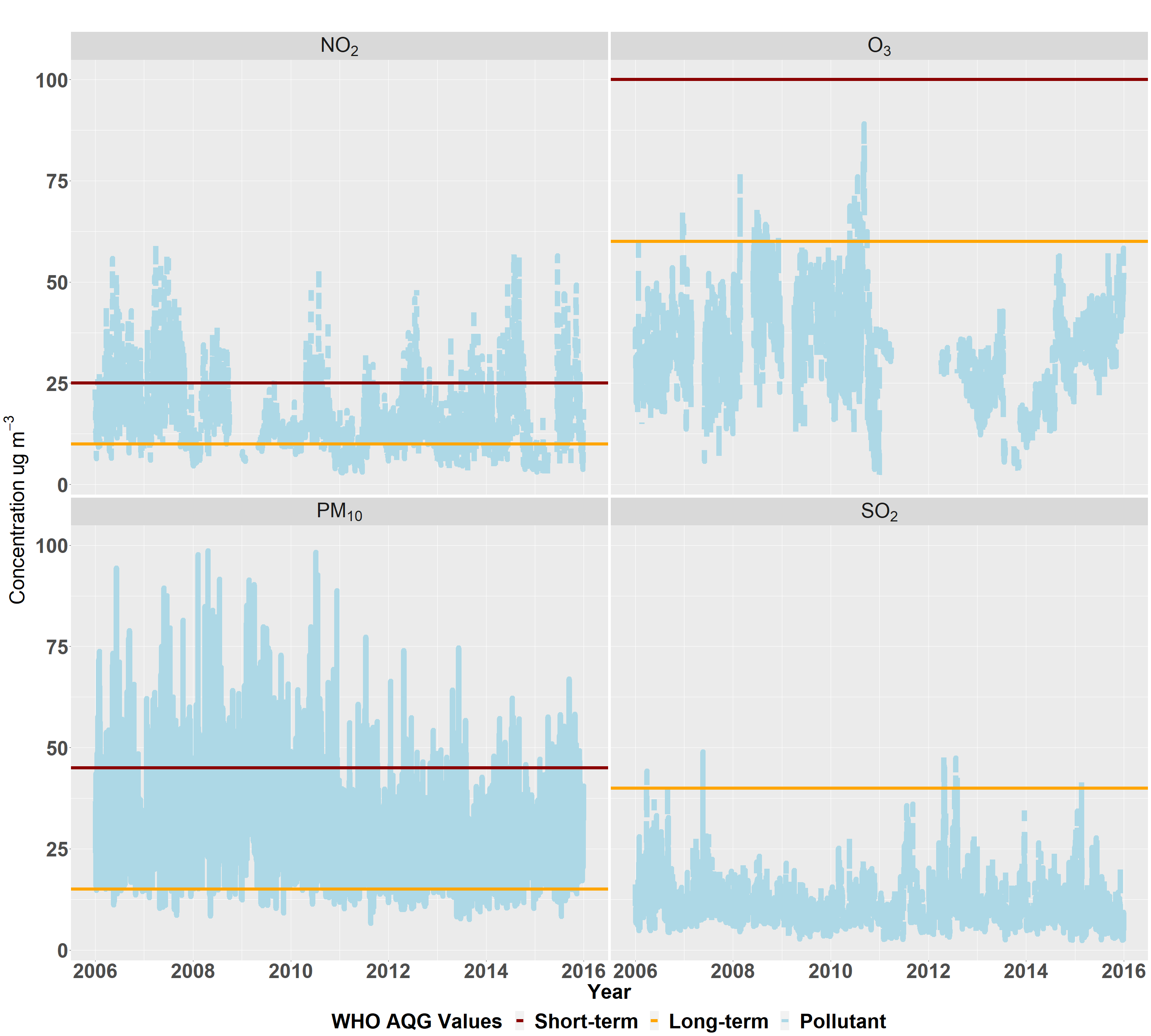


Figure S 2: Daily mean concentrations of PM10, NO2, SO2 and O3 in Cape Town, 2006 – 2015. The horizontal lines show the WHO AQG 2021 values for short-term (black) and long-term (orange) of each pollutant.

**Sensitivity analysis (**PM_10_ **versus** PM_2.5_ **based index)**

The means of the weighted average AQHI for PM_2.5_ and PM_10_ using data from 2019 (365 days) were similar, 5.6 and 5.4, respectively. There was no statistically significant difference between the daily weighted average indices using the Welch t-test, (t = 1.61, 95% CI: -0.05; 0.48). There was a strong and positive correlation (r = 0.92, p-value <0.001) between the two weighted average indices. The linear association had an intercept of 1.01 (95% CI: 0.9-1.1) and slope=0.8 as shown in the supplementary material. The two indices differed in the higher AQHI, at index 10 where the PM_2.5_-based index classified more days as high compared to the PM_10_-based index (20 vs 3). The Plots in panel A of Figure S 3 presents the daily weighted average of PM_2.5_- and PM_10_-based AQHIs; while the table in panel B shows the daily distribution of each index level for PM_2.5_ and PM_10_ based indices, where 39 and 47 days were “low risk”, respectively. The two also had a similar number of days with “moderate risk” 215 and 220, respectively, while the difference was largest in the “high risk” category, with 111 days for the PM_2.5_ based and only 98 days for the PM_10_ – based index; PM_2.5_-based index classified more days as high (index 9 and 10) compared to PM_10_-index.


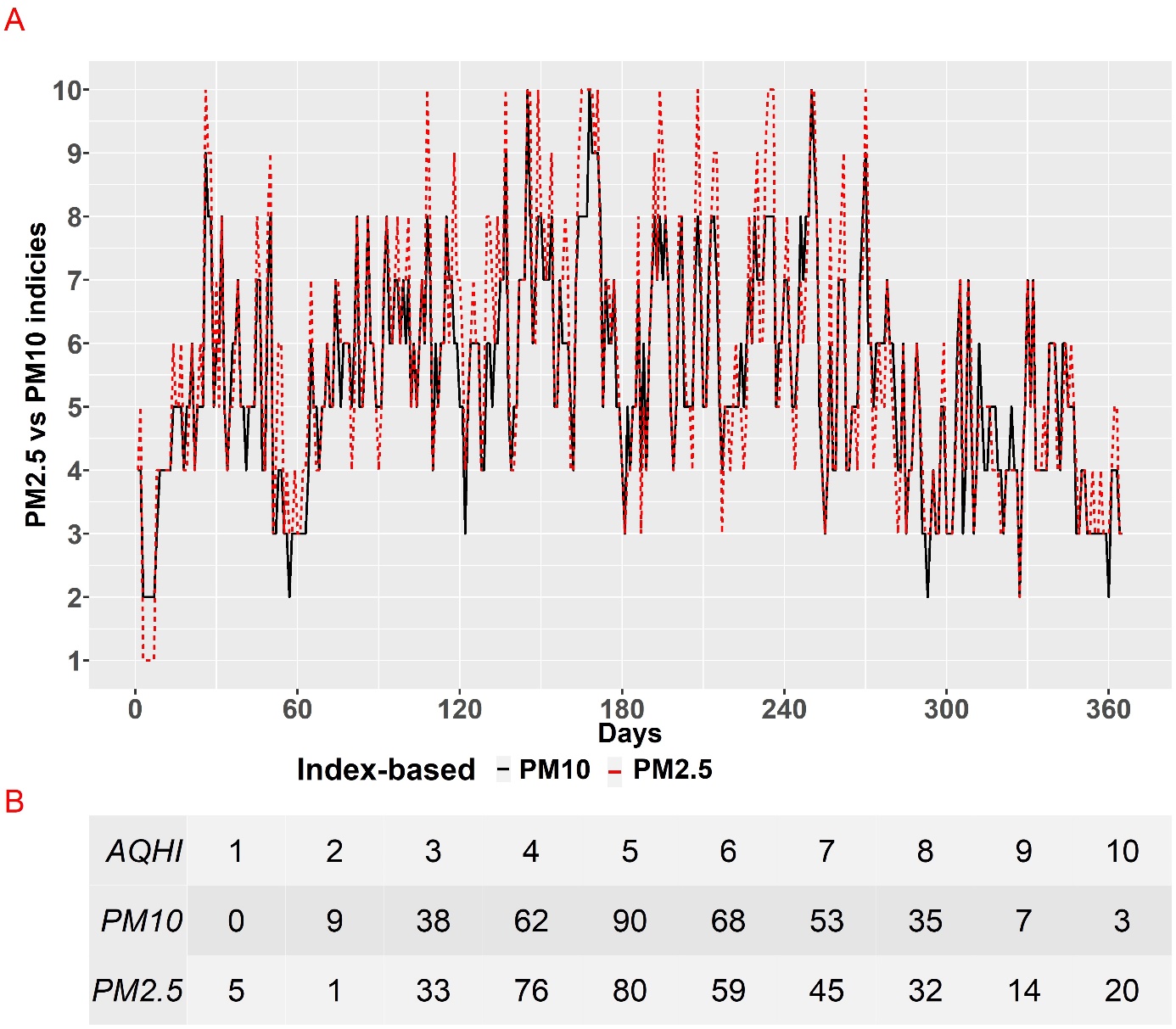


Figure S 3: Daily plot of PM10-based and PM2.5-based AQHI with the three gaseous pollutants (NO2, SO2 and O3) in 2019. Panel B shows the number of days each level of the PM10- and the PM2.5-based AQHI occurred.

This shows that one can derive an average AQHI based on either PM_10_ or PM_2.5_, whatever may be available in the monitoring networks. The question arises which one to use in case both are available. In light of the dominance of PM_2.5_ in the scientific literature and its particular dependence on anthropogenic sources of air pollution, PM_2.5_ might be the preferred choice. As shown for Cape Town, the two options lead, on average, to rather similar judgments of air quality. The observed departure from the line of identity tended towards higher index values (9 and 10+) for PM2.5 in the 14-49 µg/m3 range of pollution. The wide range is because concentrations with ER% above 1.1% for PM_2.5_ are classified as 10+. However, such distributional properties may be different in other regions and possibly depend on seasonal factors as well.

Table S 3: Distribution of daily mean (standard deviation) concentration of pollutants and number of days per average single-AQHI value in Cape Town for the period from 2006 to 2015 (in total, 3652 days)

| Single -AQHI | PM_10_ | Days | NO_2_ | Days | SO_2_ | Days | O_3_ | Days |
| --- | --- | --- | --- | --- | --- | --- | --- | --- |
| 1^[[3]](#footnote-3)^ | - | - | 3.03(0.2) | 26(0.71%) | 5.34(1.10) | 841(23.03%) | 14.39(4.51) | 374(10.24%) |
| 2 | 9.06(1.06) | 19(0.52%) | 5.29(0.97) | 255(6.98%) | 9.58(1.88) | 1948(53.34%) | 30.68(5.24) | 1612(44.14%) |
| 3 | 13.47(1.39) | 258(7.06%) | 8.46(0.95) | 460(12.6%) | 16.10(1.91) | 602(16.48%) | 47.12(5.25) | 627(17.17%) |
| 4 | 18.07(1.45) | 630(17.25%) | 11.65(0.94) | 613(16.79%) | 23.36(2.01) | 128(3.50%) | 65.29(4.58) | 54(1.48%) |
| 5 | 23.02(1.46) | 672(18.4%) | 14.84(0.94) | 596(16.32%) | 29.22(1.80) | 31(0.85%) | 83.57(3.71) | 5(0.14%) |
| 6 | 27.98(1.48) | 626(17.14%) | 18.18(0.96) | 370(10.13%) | 35.68(1.32) | 10(0.27%) | - | - |
| 7 | 32.95(1.43) | 433(11.86%) | 21.39(0.95) | 325(8.9%) | 42.71(1.88) | 10(0.27%) | - | - |
| 8 | 37.95(1.49) | 327(8.95%) | 24.68(0.96) | 219(6%) | 48.30(0.70) | 3(0.08%) | - | - |
| 9 | 42.97(1.44) | 203(5.56%) | 27.97(0.98) | 140(3.83%) | - | - | - | - |
| 10 | 57.13(10.81) | 475(13.01%) | 36.79(6.64) | 280(7.67%) | - | - | - | - |
| Missing | - | 9(0.25%) | - | 368(10.08%) | - | 79(2.16%) | - | 980(26.83%) |

Table S 4 demonstrates the derivation of the average index using an example of a typical day as observed in the Cape Town case study. The index value shown for each pollutant approximates the average index observed during the year 2015. As shown, the arithmetic mean AQHI for such a day would be 3, thus, lower than the weighted average of 4. The arithmetic mean AQHI is biased toward low values, given the mostly low index values for SO_2_ and O_3_, masking the impact of PM_10_ and NO_2_.

Table S 4: derivation of weighted average PM10-based AQHI for a typical day in Cape Town in comparison to the arithmetic average

| Pollutant | | PM_10_ | NO_2_ | SO_2_ | O_3_ | AQHI |
| --- | --- | --- | --- | --- | --- | --- |
| Single AQHI |  | 5 | 4 | 2 | 2 |  |
| Arithmetic mean AQHI | $({5+4+2+2)}/4=3.25 \sim3$ | | | | | 3 |
| Weighted Average AQHI | ${\begin{aligned} (\left( 1*5 \right)+\left( 0.85*4 \right)+\left( 0.52*2 \right)+ \\ (0.24*2)) \end{aligned}}/{2.61=3.8 \sim4}$ | | | | | 4 |

**Missing data and AQHI**

The current practice in South Africa is to derive and report the AQI for each station using measurements of available pollutants, thus the proposed new index can be derived in a similar fashion. However, authorities will face the challenge of missing pollutant data on certain days. In case of the weighted average, summary measures may be derived across the non-missing pollutants only, which in turn may lead to biased averages given the distributional properties discussed above. Alternatively, we have propose a simple imputation of missing data using measurements from other stations as this would minimize biased weighted averages. The approach uses the monthly mean of the missing station for imputation, as well as measurement data for the same pollutant at other sites.

For the sites with measurements on day t, the ratio between the respective daily mean and the last calendar monthly mean of day t is computed; ratios from these sites are then averaged. Finally, the missing daily mean is calculated by multiplying the monthly mean of the station with the missing value by the computed average ratio as shown in Equation S 2.

|  | $X_{t}= V_{t}* \frac{1}{n}\sum_{i=1,\ldots,n} \frac{D_{i,t}}{M_{i,t}}$ | Equation S 2 |
| --- | --- | --- |

$$where t=day of missing value of the respective pollutant at the given station,$$

$$X_{t}= imputed mean of pollutant at the given station on day t, V_{t}=mean of pollutant at the given station in the month of day t, D_{i,t}=mean of pollutant at station i on day t, M_{i,t}= mean of pollutant at station i in the month of day t, n=number of stations with measurements of the pollutant on day t$$

The linear regression between PM_10_- and PM_2.5_ based indices in Cape Town for 365 days had a slope of 0.78, intercept of 1.01 (95% CI: 0.9 – 1.1) and an adjusted R^2^ of 0.85.


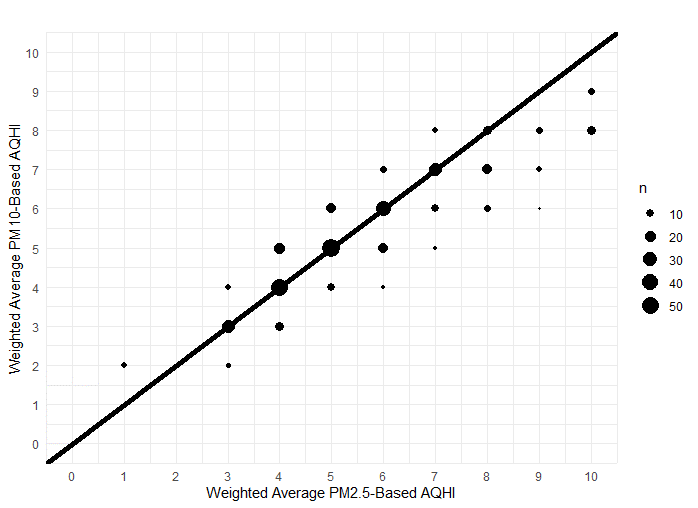


Figure S 4: Linear regression between PM_10_- and PM_2.5_ based indices in Cape Town, South Africa, 2019. Plot shows the number of days, adjusted R^2^, intercept and its 95% confidence interval and the slope with the size of points representing the number of respective days, as well as the identity line.

Table S 5: The 2021 WHO Air Quality Guideline values and South Africa’s National Ambient Air Quality Standard (NAAQS)

| Guidelines | Averaging time | PM_2.5_ | PM_10_ | NO_2_ | SO_2_ | O_3 (8-hour)_ |
| --- | --- | --- | --- | --- | --- | --- |
| WHO | Short-term^a^ | 15 | 45 | 25 | 40 | 100 |
| South Africa | Short-term | 40 | 75 | 200 (1 hr) | 125 | 120 |
|  |  |  |  |  |  |  |
| WHO | Annual | 5 | 15 | 10 | - | 60 ^b^ |
| South Africa | Annual | 20 | 40 | 40 | - | - |

^a^ Short-term is 24-hours for all pollutants (i.e. 3-4 exceedance days per year) and

^b^ Average of daily maximum 8-hour O_3_ concentrations in the six consecutive months with the highest six-month running-average O_3_ concentration.

The figure below shows the current Air Quality Index in South Africa. The concentrations for each band and their corresponding descriptions such as “good” air quality and “moderate” air quality.


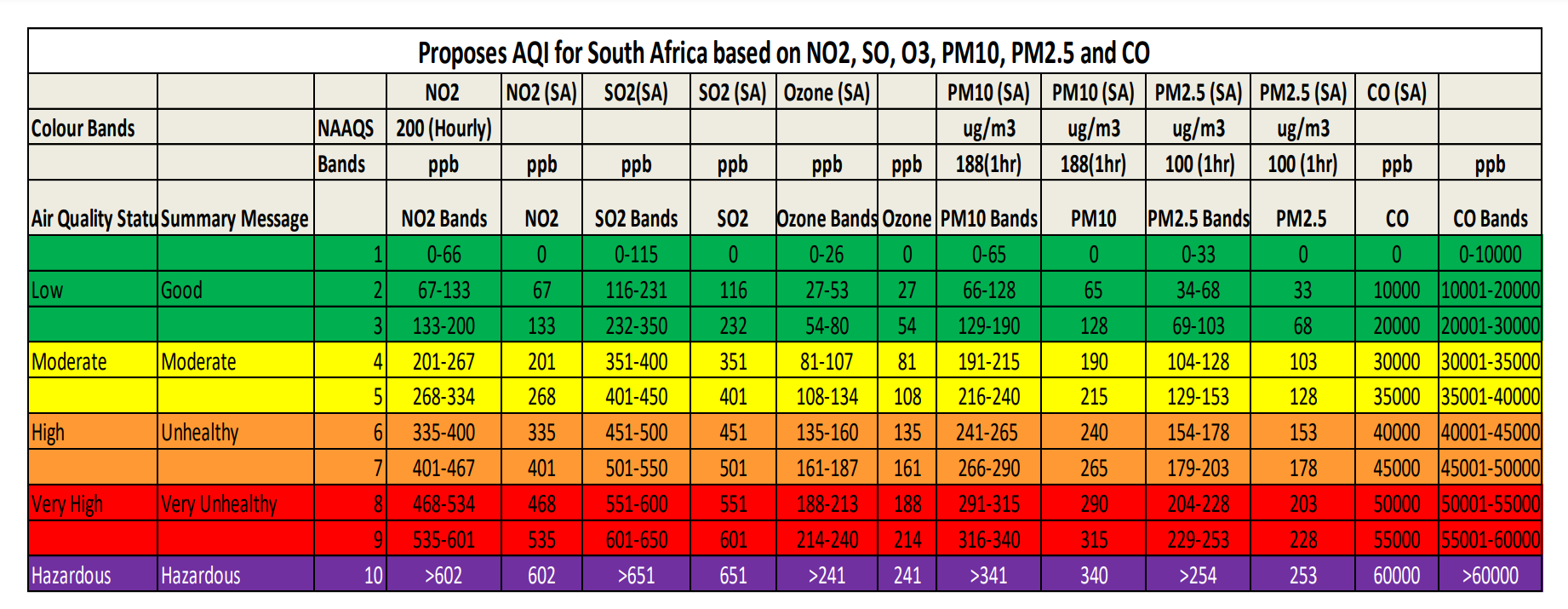


Figure S 5: South Africa AQI bands presented in 2018^11^

Parts per billion conversion to µg/m^3^ for NO_2_, SO_2_ and O_3_ using concentrations within the “Good” band of SA’s AQI:

NO_2_ 1 ppb = 1.88 µg m^-3^; SO_2_ 1 ppb = 2.62 µg m^-3^; O_3_ 1 ppb = 1.96 µg m^-3^

The figure below shows the air pollution index (API) developed by Cairncross^12^, the excess risk range for each AQI level and the corresponding risk levels.


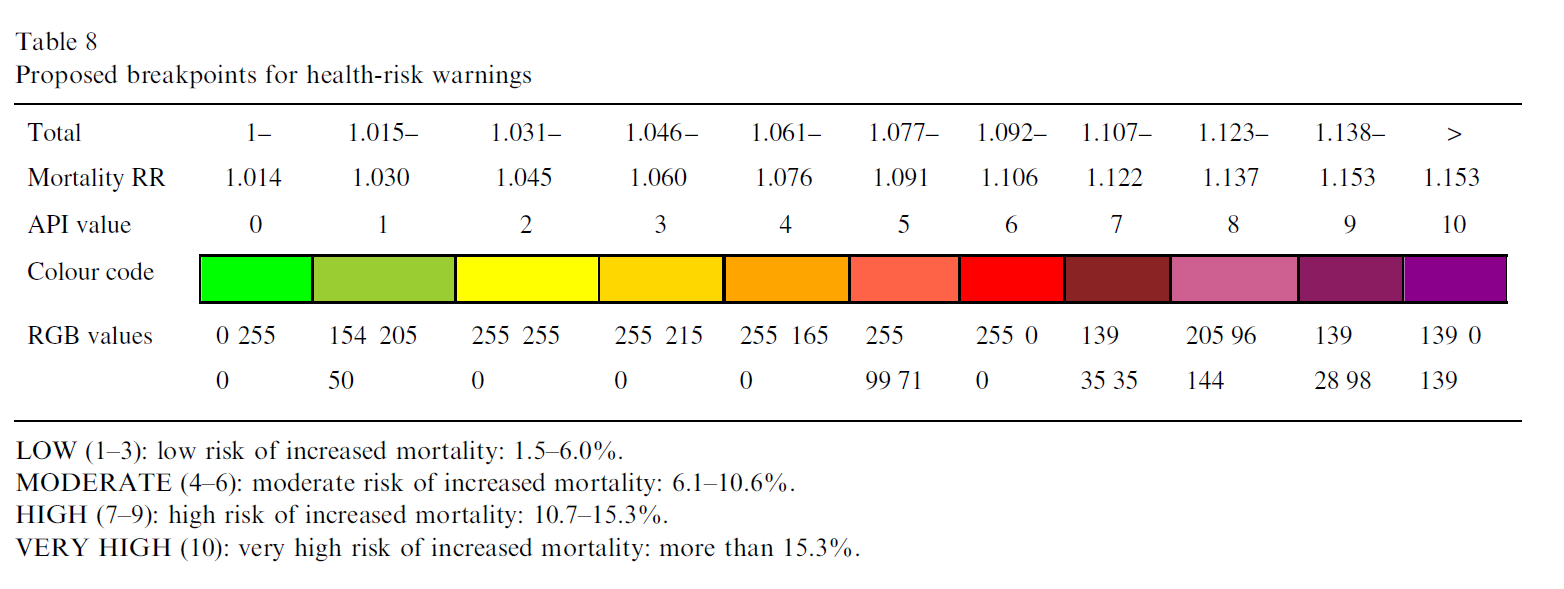


Figure S 6: API constructed by Cairncross 2007^12^

References

1. Orellano P, Reynoso J, Quaranta N, Bardach A, Ciapponi A. Short-term exposure to particulate matter (PM10 and PM2.5), nitrogen dioxide (NO2), and ozone (O3) and all-cause and cause-specific mortality: Systematic review and meta-analysis. *Environment International.* 2020;142:105876.

2. Orellano P, Reynoso J, Quaranta N. Short-term exposure to sulphur dioxide (SO(2)) and all-cause and respiratory mortality: A systematic review and meta-analysis. *Environ Int.* 2021;150:106434.

3. Liu C, Chen R, Sera F, et al. Ambient Particulate Air Pollution and Daily Mortality in 652 Cities. *N Engl J Med.* 2019;381(8):705-715.

4. Vicedo-Cabrera AM, Sera F, Liu C, et al. Short term association between ozone and mortality: global two stage time series study in 406 locations in 20 countries. *Bmj.* 2020;368:m108.

5. World Health Organization. *WHO global air quality guidelines: particulate matter (PM2.5 and PM10), ozone, nitrogen dioxide, sulfur dioxide and carbon monoxide.* Geneva: World Health Organization; 2021.

6. World Health Organization. Regional Office for Europe. *Air quality guidelines for Europe.* 2nd ed. ed. Copenhagen: World Health Organization. Regional Office for Europe; 2000.

7. United Nations Economic Commission for Europe. Manual on methodologies and criteria for Modelling and Mapping Critical Loads & Levels and Air Pollution Effects, Risks and Trends. Chapter III on Mapping Critical Levels for Vegetation (version 2017). In:2017.

8. National Air Quality Indicator - Monthly data report for the Western Cape Province. 2022. <https://saaqis.environment.gov.za/>. Accessed 09 May 2022.

9. Héroux ME, Anderson HR, Atkinson R, et al. Quantifying the health impacts of ambient air pollutants: recommendations of a WHO/Europe project. *Int J Public Health.* 2015;60(5):619-627.

10. Stieb DM, Burnett RT, Smith-Doiron M, Brion O, Shin HH, Economou V. A new multipollutant, no-threshold air quality health index based on short-term associations observed in daily time-series analyses. *J Air Waste Manag Assoc.* 2008;58(3):435-450.

11. Gwaze P, Mashele SH. South African Air Quality Information System (SAAQIS) mobile application tool: Bringing real time state of air quality to South Africans. *Clean Air Journal.* 2018;28(1):3-3.

12. Cairncross EK, John J, Zunckel M. A novel air pollution index based on the relative risk of daily mortality associated with short-term exposure to common air pollutants. *Atmospheric environment.* 2007;41(38):8442-8454.

1. The ambient concentration for each pollutant are the WHO long-term AQG values. [↑](#footnote-ref-1)
2. WHO 2021 AQG did not include a long-term value for SO2, thus we used the value from the 2000 WHO AQG. The rationale for this is provided in the “derivation of excess risk” section of the method [↑](#footnote-ref-2)
3. Empty cells show there are no ER% calculated for those days, either because the data was missing or the pollutant concentrations did not fall with the ER% range for the AQHI [↑](#footnote-ref-3)
